# Using district health information to monitor sustainable development

**DOI:** 10.2471/BLT.19.239970

**Published:** 2019-11-29

**Authors:** Andrea Farnham, Jürg Utzinger, Alexandra V Kulinkina, Mirko S Winkler

**Affiliations:** a Swiss Tropical and Public Health Institute, Socinstrasse 57, CH-4051 Basel, Switzerland.; b Partners In Health, Neno, Malawi.

Timely access to quality data is a key aspect of global governance and accountability. Data on development and health indicators are important for policy-makers, public health experts and donors. With the endorsement of *Transforming our world, the 2030 agenda for sustainable development*, with its 17 sustainable development goals (SDGs) and their 232 indicators, the demand for data at all levels has increased. This demand is placing pressure on national monitoring and reporting systems, particularly in low- and middle-income countries.

The final assessments of country-level progress in global health achieved between 2000 and 2015 were often based on sparse or outdated data, leading to misplaced confidence in the results.^1^ Some of these data were collected five years ago or more, leading to a considerable potential for incorrect conclusions and thus ineffective policy decisions. Therefore, the long-term solution to adequately track progress towards the SDGs is investment in the production of empirical data through national health management information systems, instead of reliance on out-dated estimates. An adequate health management information system that allows close monitoring of population health through the systematic collection of data from health facilities nationwide is a key building block of national health system planning and decision-making.

## Integrating priorities

Health management information systems data will only be adequate to track progress towards the SDGs and other national goals when reporting coverage and data quality are consistently high, as well as aggregated into meaningful internationally agreed upon indicators. Although large quantities of data are currently produced in low- and middle-income countries, these data are often of poor quality, unable to be integrated with other information systems, and only indirectly related to the indicators that are important for the SDGs and national priority setting. Many factors contribute to the issues of data quality and completeness, such as siloed data systems caused by donors’ mandates^2^ and the lack of integration of data from the recent explosion of digital health interventions. The issue of insufficient, inaccurate or outdated data is particularly salient on the African continent, where tracking progress on the 17 SDGs is pressing.

The District Health Information System 2 (DHIS2) platform has been portrayed as a solution to many of these problems. This platform was developed by Health Information Systems Programme and is supported by the Norwegian Agency for Development Cooperation, the United States President’s Emergency Plan for AIDS Relief, the Global Fund to Fight AIDS, Tuberculosis and Malaria, the United Nations Children’s Fund and the University of Oslo. This platform is an open-source data collection and management tool that provides users with a flexible interface for managing health data. In addition to data collection and management capabilities, the platform has built-in data validation, visualization and analysis tools, allowing end-users to access and analyse health data at all levels of the health system. The use of electronic forms for data collection provides for more efficient and accurate collation of data at the national level with better quality control measures. 

Since the initial rollout of the online version of DHIS2 in Kenya in 2011, the platform has been used in 40 countries in Africa. To evaluate the impact of the platform’s data on African research output, we examined the peer-reviewed literature to assess the extent to which the data have been successfully used to monitor country-level health indicators.

## Peer-reviewed literature

**Fig. 1:**
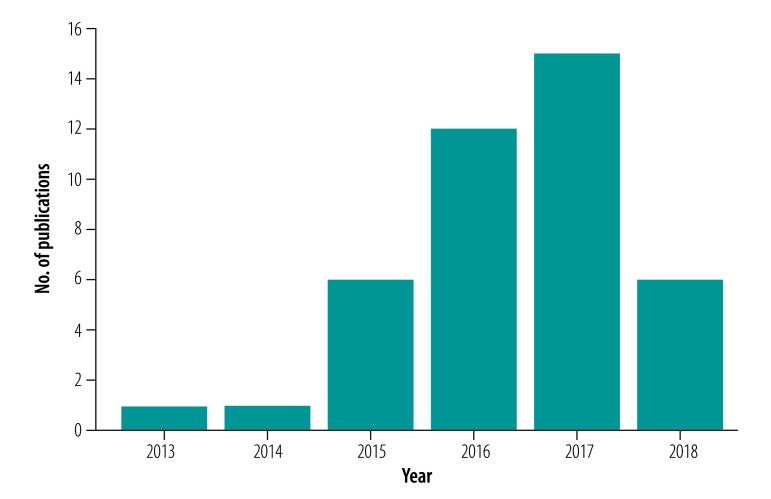
Identified studies using data from the District Health Information System 2, Africa, 2013–2018

Note: We searched Scopus, Web of Science, and PubMed® for studies published between 2011 and 29 April 2019 on all permutations of District Health Information System 2.

A search of all permutations of DHIS2 between 2011 and 29 April 2019 in Scopus, Web of Science and PubMed® found a total of 41 articles that employed the platform’s data to analyse disease trends or assess the impact of an intervention within Africa. The papers identified used data from Kenya (nine studies), Zambia (nine studies), Nigeria (six studies), Uganda (five studies), Zimbabwe (three studies), Ghana (two studies), Liberia (two studies), United Republic of Tanzania (two studies), Malawi (one study) and Rwanda (one study). There was one Pan-African paper. South African publications were excluded due to incomplete implementation of the online version of the platform. The largest number of studies used malaria data (17 studies), followed by maternal and child health data (12 studies) and human immunodeficiency virus data (six studies). An additional 35 papers identified described the platform’s implementation or post-implementation data quality in Africa. The peer-reviewed literature employing the platform’s data for analysis increased from only two articles in 2013 and 2014 to six articles in 2015, 12 articles in 2016, and 15 articles in 2017 (Fig. 1). Interestingly, only six articles were published in 2018. While we expect this search was not exhaustive and may have missed papers that did not specifically reference the platform or used a different local name for the platform, we believe the conclusions we draw are representative.

The identified articles showed that the results of the platform’s implementation varied. Several studies point to measurable improvements in data quality and quantity after implementation, along with wide acceptance and positive user experience.^3^^,^^4^ In other countries, researchers were able to measure changes in key health indicators over time, evaluate the impact of public health interventions and vaccine campaigns, and track disease outbreaks using the platform’s data.^5^^,^^6^ In Liberia, for example, data were used to describe major changes in population birth rates after the 2013–2016 Ebola virus disease outbreak in West Africa.^7^ In the United Republic of Tanzania, specific workshops to involve feedback from local platform users improved subsequent data quality and coverage.^8^

Although these early experiences show promise, other studies indicate that concerns remain. Usually data are still collected in health facilities by often-overworked health-care workers, and only later manually entered into the platform, introducing potential delays and errors.^3^^,^^9^ Moreover, data quality is not uniform across indicators, and private-sector reporting often lags behind that of the public sector .^3^^,^^4^ While district-level training on the platform is often comprehensive, it is inadequate or non-existent at the facility level.^9^^–^^11^ Data entry continues to be a unidirectional process, with health-facility staff often unaware of the purpose of the data they are collecting and performing only limited data quality checks.^11^^,^^12^ Finally, internet connectivity continues to be a challenge in many settings, meaning that the platform’s database is only intermittently accessible at the local level.

## Monitoring of the SDGs

The results from the identified articles indicate that the platform has improved the data quality and quantity available from the health management and information systems in most African countries. However, the relative lack of high-quality publications using these data is troubling. Many African countries are sitting on an enormous untapped data resource; after national health systems policy-makers made significant efforts to collect and integrate these health data into national databases, many African countries will fail to realize the full potential of the platform if the resulting data remain unanalysed and unpublished.

The reasons for this lack of publication are multifaceted. First, the research and analysis capacity to synthesize and publish the data is scarce. Second, the institutional culture does not promote routine use of the platform’s data for programme management and decision-making. Third, data quality across indicators is inconsistent. The first two issues can be addressed by investment in capacity building of end-users and prioritization of data science education at the national level, within the health system. However, data quality is the most challenging obstacle to promoting the use of the platform for tracking SDG indicators across Africa. The quality of indicators that donors prioritize is often higher than that of other indicators that are equally important for monitoring population health and progress towards the SDGs.^9^ This prioritization of data by donors is reflected in the dominance of malaria data in the papers based on the platform’s data published so far. Additionally, reporting burden is often high at the local level,^11^ reducing data quality and causing duplication of efforts. The streamlining of the reporting burden and emphasis on the indicators must be coordinated centrally and in collaboration with national and international experts to determine which indicators are important for meeting national and international goals.

For the platform to reach its full potential in measuring progress towards the SDGs, the data must be standardized, accessible and transparent. Furthermore, countries should clearly define and prioritize indicators that can be reliably measured via the platform. For instance, a combination of adequate resources to diagnose and treat a specific disease and high-service use measured at the facility level can serve as a reliable proxy for the burden of this disease. Meanwhile, for morbidity and mortality estimates for a disease that does not have universally available diagnostic tests, or for deaths happening at home, the platform has clear limitations. In our view, the platform’s data has been so far underused. Beyond the 2030 agenda, this underuse is a lost opportunity for countries to use their own data to create national health agendas, measure the impact of health interventions and investigate the burden of diseases not prioritized by donors. However, the foundation of a robust national health management information system is now in place in most low- and middle-income countries. This system should become the primary source of quality data for resource allocation and impact measurement. In building upon this foundation, investing in the entire health system, including human resources, infrastructure and supplies, and the database itself, is required to lead to reliable platform data across health areas. Public health experts should take advantage of the synergies in the SDGs and their national priorities to change health and management information systems in Africa.
